# Cancer-associated fibroblasts expressing fibroblast activation protein and podoplanin in non-small cell lung cancer predict poor clinical outcome

**DOI:** 10.1038/s41416-024-02671-1

**Published:** 2024-04-06

**Authors:** Layla Mathieson, Lilian Koppensteiner, David A. Dorward, Richard A. O’Connor, Ahsan R. Akram

**Affiliations:** 1 Centre for Inflammation Research, Institute of Regeneration and Repair, University of Edinburgh, 5 Little France Dr, Edinburgh BioQuarter, Edinburgh, EH16 4UU UK; 2 Translational Healthcare Technologies Group, Centre for Inflammation Research, Institute of Regeneration and Repair, University of Edinburgh, 5 Little France Dr, Edinburgh BioQuarter, Edinburgh, EH16 4UU UK; 3 Department of Pathology, Royal Infirmary of Edinburgh, Edinburgh, UK; 4 Cancer Research UK Scotland Centre, Institute of Genetics & Cancer, The University of Edinburgh, Crewe Road South, Edinburgh, EH4 2XR UK

**Keywords:** Non-small-cell lung cancer, Fluorescence imaging, Flow cytometry

## Abstract

**Background:**

Cancer-associated fibroblasts (CAFs) are a dominant cell type in the stroma of non-small cell lung cancer (NSCLC). Fibroblast heterogeneity reflects subpopulations of CAFs, which can influence prognosis and treatment efficacy. We describe the subtypes of CAFs in NSCLC.

**Methods:**

Primary human NSCLC resections were assessed by flow cytometry and multiplex immunofluorescence for markers of fibroblast activation which allowed identification of CAF subsets. Survival data were analysed for our NSCLC cohort consisting of 163 patients to understand prognostic significance of CAF subsets.

**Results:**

We identified five CAF populations, termed CAF S1-S5. CAF-S5 represents a previously undescribed population, and express FAP and PDPN but lack the myofibroblast marker αSMA, whereas CAF-S1 populations express all three. CAF-S5 are spatially further from tumour regions then CAF-S1 and scRNA data demonstrate an inflammatory phenotype. The presence of CAF-S1 or CAF-S5 is correlated to worse survival outcome in NSCLC, despite curative resection, highlighting the prognostic importance of CAF subtypes in NSCLC. TCGA data suggest the predominance of CAF-S5 has a poor prognosis across several cancer types.

**Conclusion:**

This study describes the fibroblast heterogeneity in NSCLC and the prognostic importance of the novel CAF-S5 subset where its presence correlates to worse survival outcome.

## Background

Lung cancer is the leading cause of cancer death globally [1] and non-small cell lung cancer (NSCLC) accounts for ~85% of cases [2]. Current NSCLC therapies are often unsuccessful, with drug resistance leading to treatment failure and disease progression [3]. The tumour stroma plays a role in this resistance to therapy and has emerged as an important target for therapies to combat cancers such as NSCLC [4–7].

One of the most common cell types of the tumour stroma is the cancer-associated fibroblast (CAF) [8]. In health, fibroblasts are a quiescent structural component of the extracellular matrix (ECM), which become activated in response to wound signalling. In their activated state they produce ECM components, and engage in crosstalk with immune cells to promote wound healing [9]. CAFs are irreversibly activated, have an enhanced migratory phenotype over normal activated fibroblasts, a greater proliferative ability and an enhanced secretome [10]. CAFs also have roles in immune evasion, metastasis, invasion, angiogenesis and resistance to drug treatment [6, 11–15].

Several studies have shown that CAFs represent a heterogeneous population composed of functionally distinct subtypes [6, 16–21]. The phenotype of these subtypes has been characterised in some solid organ malignancies, including breast, ovarian, pancreatic and lung cancers [17, 19–23]. Markers frequently used to distinguish these subtypes include α-smooth muscle actin (αSMA), fibroblast activation protein (FAP), podoplanin (PDPN), integrin β1 (CD29) and fibroblast-specific protein-1 (FSP-1). Two key subtypes of note, commonly termed CAF-S1 and CAF-S4, have been identified in several studies, CAF-S1 display a FAP-hi phenotype associated with adhesion, wound healing and immunosuppression while CAF-S4 which are FAP-low/negative and express higher levels of αSMA are more contractile and associated with invasion and metastasis [7, 17, 20, 24–27]. Spatially, CAF-S1 have been found in closer proximity to cancer cells. The presence of these subtypes can also indicate prognosis, with CAF-S1 and CAF-S4 being found to promote metastases, and CAF-S1 being an indicator of distant relapse in luminal breast cancer [20].

Here, we investigate CAF subtypes present in NSCLC, identifying five subtypes using commonly used CAF markers. We focus on the novel CAF-S5 subtype, identified primarily by the expression of FAP and PDPN but lacking expression of αSMA. We compare the spatial location of CAF-S5 to the previously defined CAF-S1 subtype and investigate the correlation of these subtypes to survival outcome.

## Methods

### NSCLC patient sample processing

Fresh excised tumour and adjacent non-cancerous lung (NCL) tissues were collected following surgical resection, where NCL tissues were taken from the most distal point in the same lobe as the tumour. Samples were stored in DMEM (Gibco) containing 100 U/L penicillin-streptomycin (Gibco) and processed within 16 h. Tissue samples were minced with forceps and incubated in a water bath at 37 °C for an hour in prewarmed RPMI media (Gibco) containing collagenase I [1 mg/ml] (Gibco) and DNase [0.1 mg/ml] (Sigma), with samples removed for vortexing every 10 min within that incubation. Samples were then centrifuged at 300 × *g* for 5 min and supernatant removed, then 5 ml TrypLE express (Gibco) was added. Samples were incubated for a further 5 min at 37 °C, before being centrifuged at 300 × *g* for 5 min and supernatant removed. Samples were then resuspended in media and passed through a 70 μm cell strainer and red blood cells were lysed from samples using RBC lysis buffer (Roche) in 5 ml for 10 min at room temperature. Cells were washed in plain RPMI media and then counted in preparation for staining.

### Flow cytometry sample preparation

Following tissue digest, cells were resuspended in DPBS (Gibco) for staining by flow cytometry, with 1 million cells per condition. For all conditions other than the unstained control, cells were stained with a live/dead marker Zombie UV (1:1000, Biolegend) for 30 min at room temperature in DPBS (Gibco). Cells were then washed (centrifuged at 300 × *g* for 5 min) in DPBS supplemented with 2% FBS (FACs buffer) and incubated with FC blocker (Biolegend) for 10 min and then stained with surface marker antibodies (EpCAM, CD45, CD31, FAP, CD29, Podoplanin and PDGFRβ, see Supplementary Table S1 for details) or the corresponding isotype control antibodies for 20 min at 4 °C in FACs buffer. After washing cells were fixed with Cytofix fixation buffer (BD Biosciences) for 20 min at 4 °C. Cells were then washed in Perm/Wash buffer (BD Biosciences) and centrifuged at 300 × *g* for 5 min. Intracellular antibodies (αSMA and FSP-1) or the corresponding isotype controls were diluted in Perm/Wash buffer then added to cells and incubated in the dark for 30 min at 4 °C. After washing, cells were stored in DPBS with 2% FBS overnight at 4 °C before data acquisition on a LSR6Fortessa analyser (BD Biosciences). Compensation was carried out using single stain control UltraComp eBeads (Invitrogen).

### Flow cytometry data analysis

Flow cytometry data were analysed using FlowJo version 10.7.1. Cells were gated to fibroblast populations defined as CD45^−^, EpCAM^−^ and CD31^−^ cells (full gating strategy shown in Fig. S1). To reduce file sizes for analysis, fibroblast populations were downsampled to 300 events using the Downsample plugin. Samples containing less than 300 fibroblasts were excluded from analysis. All sample files were then concatenated and from this file UMaps could be generated from the data [28]. FlowSOM analysis could then be carried out to determine clusters and was run without defining the number of clusters expected to be unbiased [29]. MFIs calculated were the geometric fluorescence intensity.

### TMA generation

A TMA was constructed as previously described [30, 31] from tumour resections from patients undergoing surgery for treatment of NSCLC with curative intent. Here a total of 163 cancer cores were available for staining.

### Multiplex immunofluorescence staining

Slides were deparaffinised in Xylene and rehydrated in a series of ethanol dilutions. Using the Leica Bond automated staining robot, samples underwent heat-induced antigen retrieval (HIER) of 30 min at 100 °C. Then tissue slides were exposed to multiple staining cycles each including a 30 min incubation with a protein block (Akoya), 1 h incubation with the respective primary antibody, 30 min incubation with the secondary antibody (Akoya), 10 min incubation with the respective OPAL (Akoya) followed by 20 min incubation with AR6 buffer (Akoya) at 85 °C prior to the next staining cycles and finally stained with fluorescent DAPI (Akoya) for 10 min. In between each step, slides were washed with bond wash for 5 min.

Primary antibody concentrations and OPAL pairings are shown in Supplementary Table S2. Antibody-OPAL pairings were assigned based on expected biomarker abundance and expected co-expression. Dilution of antibodies was assessed by single stains.

### Multiplex immunofluorescence imaging

Slides were imaged using a Vectra Polaris. The appropriate exposure time for image acquisition was set for each fluorophore by auto exposing on multiple (5–10) tissue areas per batch. Following fluorescence whole slide scans, regions of interest were selected for multispectral imaging (MSI) at ×20 magnification.

### Multiplex immunofluorescence image analysis

MSI images were unmixed in InForm software using representative snapshots of spectral library slides imaged at the same magnification. This also allowed for the isolation of auto fluorescence. Unmixed images were exported and analysed in Qupath [32]. Cell detection was performed using StarDist based on a watershed deep-learning algorithm and fluorescent threshold of DAPI nuclear staining [33]. Following this, phenotyping was performed in a non-hierarchical manner by creating a composite classifier of single channel classifiers for each stain based on a fluorescent threshold that applied across the whole tumour collection. Ultimately, a machine learning algorithm was trained on multiple images to detect tumour and stroma areas. For each image the counts of the number of cells classified by each combination of markers was calculated and exported for analysis using R. Using the definitions established by flow cytometry to characterise a profile for each subset as having markers on or off we defined subsets as: CAF-S1: FAP^ON^ αSMA^ON^ FSP1^OFF^ CD90^ON^ PDPN^ON^, CAF-S4: FAP^OFF^ αSMA^ON^ FSP1^OFF^ CD90^ON^ PDPN^OFF^, CAF-S5: FAP^ON^ αSMA^OFF^ FSP1^OFF^ CD90^OFF^ PDPN^ON^. This binary classification allowed for classification of individual cells as each subtype.

### Single cell RNA sequencing analysis

Open source data from Lambrechts et al. [22] and Wu et al. [34] were analysed using R. Lambrechts et al. data (referred to as early NSCLC due to patients being those classed as untreated, primary, non-metastatic cases who were undergoing surgery with curative intent) were downloaded from https://scope.aertslab.org/#/Bernard_Thienpont/Bernard_Thienpont%2FThienpont_Fibroblast_v4_R_fixed.loom/gene and Wu et al. data (late NSCLC) from the gene expression omnibus under GSE148071. The fibroblast data sets were filtered for fibroblasts that could be defined as CAF-S1 or CAF-S5 using the definitions of the subtypes established by flow cytometry. Fibroblasts were filtered by including those with expression of CD29, PDGFRβ, PDPN and FAP and excluding any that expressed FSP1. The remaining fibroblasts were then determined to be CAF-S1 if they expressed αSMA and CAF-S5 if they did not express αSMA. Differential expression analysis was then performed in R using the DESeq2 package [35]. The top 50 differentially expressed genes were plotted in a heat map and volcano plots of all genes were generated to assess key differentially regulated genes between the two subtypes.

### Analysis of survival data

Survival data was collected for the 163 NSCLC patients whose samples were included in the TMA analysed by multiplex immunofluorescence, where overall survival was defined as the number of days from diagnosis to death and recurrence free survival defined as the number of days from diagnosis to death or recurrence. Kaplan–Meier curves were plotted for patients who had fibroblasts of phenotype CAF-S1 or CAF-S5 present (determined in QuPath as described above) above and below the median number of CAFs present in that subtype. Log-rank tests were used to determine significance. Plots were also generated for the markers FAP, PDPN and αSMA, showing survival when these markers are present above or below median expression levels. Analysis was carried out using the survival and survminer packages in R.

### Analysis of TCGA data

Data for liver hepatocellular carcinoma (TCGA-LIHC), pancreatic adenocarcinoma (TCGA-PAAD), invasive breast carcinoma (TCGA-BRCA) and renal clear cell carcinoma (TCGA-KIRC) were downloaded from https://tcga-data.nci.nih.gov. The surv_cutpoint function in R was used to determine the most significant cut off for expression level correlated to survival for each cancer for the markers FAP, PDPN and αSMA. Using these cut-offs generated patients could be defined as low or high for each marker. Patients were defined to have an overall CAF-S5 phenotype if they were FAP and PDPN high and αSMA low. The survival of these patients was then compared all other patients by plotting Kaplan–Meier curves as described above.

### Statistical analysis

All scatter, violin and boxplots were plotted and statistical testing performed using GraphPad Prism version 9. Error bars represent the standard deviation. For the flow cytometry data significance was determined using unpaired *t*-tests. For the multiplex immunofluorescence analysis Tukey’s multiple comparisons tests were performed. Significance was considered significant when the *p* value was <0.05 (*p ≤ 0.05, **p ≤ 0.01, ***p ≤ 0.001, ****p ≤ 0.0001). All *n* numbers are shown in the figure legends.

## Results

### CAFs in NSCLC express high levels of fibroblast activation markers

**Fig. 1:**
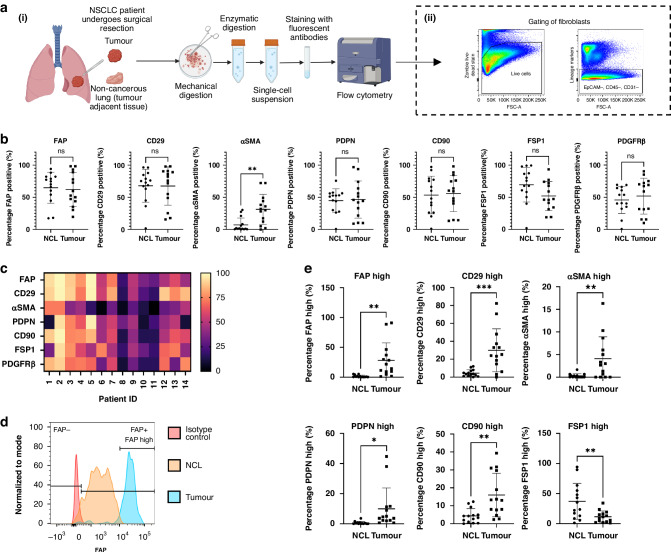
Identifying CAFs in NSCLC by expression of fibroblast activation markers. **a** The preparation of NSCLC samples for analysis of CAFs from NSCLC patient resections for analysis by flow cytometry (i) and subsequent gating strategy to define fibroblasts (ii); **b** Expression levels of FAP, CD29, αSMA, PDPN, CD90, FSP1 and PDGFRβ shown as percentage positivity of each marker per sample, determined by FACS in non-cancerous lung tissue compared to tumour tissue; **c** Heat map demonstrating the percentage positivity of each marker in tumour tissue for each patient evaluated in the study; **d** Representative plot for FAP expression showing the definition of FAP positive and negative expression and high marker expression; **e** Percentage of fibroblasts expressing high levels of FAP, CD29, αSMA, PDPN, CD90 and FSP1 in each sample comparing non-cancerous lung and tumour tissue. Individual data points shown (tumour *n* = 14, non-cancer *n* = 14) as well as mean ± SD. Unpaired *t*-test carried out as not all data points paired, *p < 0.05, **p < 0.01, ***p < 0.005.

To understand the heterogeneity of CAFs in human NSCLC we first looked at the expression levels of seven markers used to characterise CAFs using flow cytometry (Fig. 1a(i)). Fibroblasts were identified as being negative for EpCAM, CD45 and CD31 to exclude epithelial, hematopoietic and endothelial cells respectively (Fig. 1a(ii)). Fibroblast activation markers FAP, CD29, αSMA, PDPN, CD90, FSP1 and PDGFRβ expression levels were determined and compared for tumour and non-cancerous adjacent lung tissue from NSCLC patients by looking at the percentage positivity for each marker (Fig. 1b). αSMA expression was significantly upregulated in CAF. There was significant heterogeneity in marker expression between patient samples (Fig. 1c). Detailed analysis revealed differences in the levels of marker expression between fibroblasts from tumour and non-cancerous lung (NCL) tissues (Fig. 1d, e, representative plots shown for all individual markers in Fig. S2). Assessing the percentage of fibroblasts highly expressing each marker revealed significant differences between fibroblasts isolated from NCL and tumour. FAP, CD29, αSMA, PDPN and CD90 were all upregulated in tumour fibroblasts, while FSP1 was more highly expressed in NCL fibroblasts.

### Five subsets of CAFs were identified in NSCLC

**Fig. 2:**
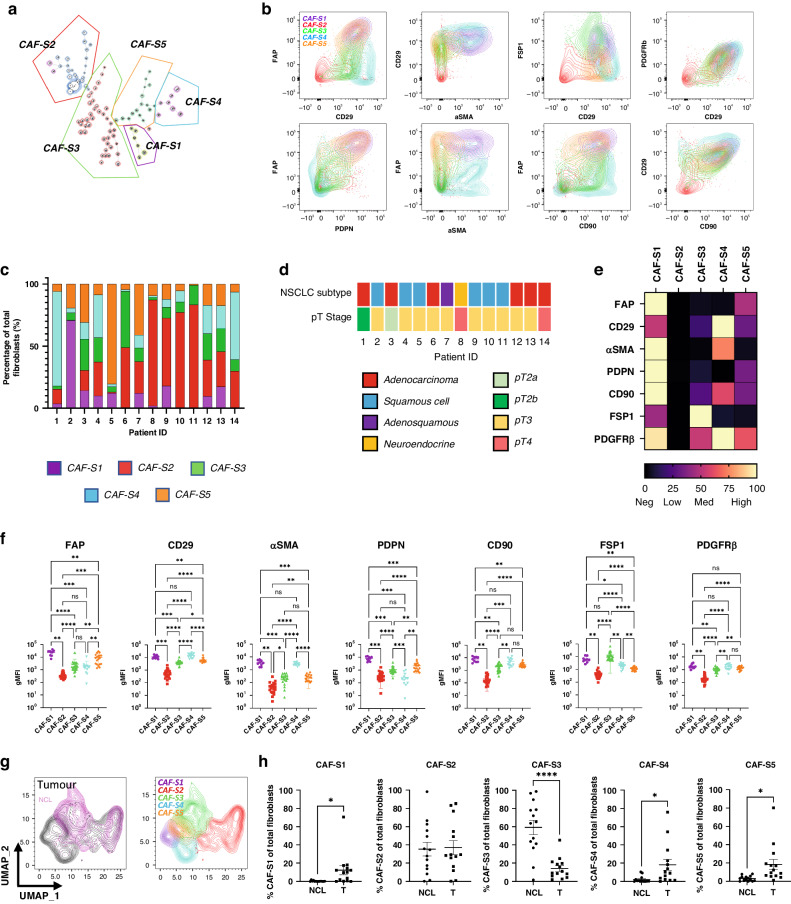
CAF subsets identified in NSCLC. **a** FlowSOM plot showing identification of five CAF subsets in NSCLC, CAF-S1–S5; **b** Expression profiles of the identified CAF subsets using the different CAF markers; **c** Breakdown of CAF subsets in individual NSCLC tumour samples; **d** NSCLC subtype and pT stage for tumour samples used in the analysis; **e** Heat map showing the relative levels of expression of each CAF marker, normalised within each marker, between identified subsets; **f** The expression levels of each marker within each subset. Each point represents geometric MFI of that marker for each sample that contained CAFs of that subset. Stats show Tukey’s multiple comparisons test results (*p ≤ 0.05, **p ≤ 0.01, ***p ≤ 0.001, ****p ≤ 0.0001); **g** UMAPs showing the clustering of the CAF subsets and the comparison of tumour and non-cancerous fibroblasts showing overlap within some subsets; **h** Comparison of the percentage of each CAF subset present in non-cancerous lung tissue (NCL) with tumour tissue. *p* values calculated using unpaired *t-*test (*p ≤ 0.05, **p ≤ 0.01, ***p ≤ 0.001, ****p ≤ 0.0001), *n* = 14 NCL and *n* = 14 tumour.

To describe CAF heterogeneity within the tumour fibroblasts FlowSOM [29] was used to determine phenotypic clusters of CAFs in an unbiased manner. This identified five subsets of CAFs across the samples (Fig. 2a), named CAF-S1 (pink), CAF-S2 (red), CAF-S3 (green), CAF-S4 (blue) following conventions set by previous studies in breast and ovarian cancers [20, 21, 36] as well as a previously unreported CAF-S5 (orange). CAF subtypes were defined by multiple markers (Fig. 2b). Mapping the distribution of CAF subsets across tumour samples (Fig. 2c), we see that the subsets are not patient specific, rather that CAF heterogeneity exists within patients. This is not driven by NSCLC subtype or stage, as the subset distribution shows no patterns evident of this when comparing to patient sample information (Fig. 2d, full patient information shown in Supplementary Table S3).

The relative expression of each marker was visualised, across subsets (Fig. 2e) and within patients (Fig. 2f). These (along with the expression profiles in Fig. S3) were used to classify the subsets as having the following expression levels:

CAF-S1: FAP^High^ CD29^Med-High^ αSMA^High^ PDPN^High^ CD90^Med-High^ FSP1^Low^ PDGFRβ^Med^,

CAF-S2: FAP^Neg^ CD29^Neg-Low^ αSMA^Neg^ PDPN^Neg^ CD90^Neg^ FSP1^Neg^ PDGFRβ^Neg^,

CAF-S3: FAP^Low^ CD29^Med^ αSMA^Neg-Low^ PDPN^Low^ CD90^Low^ FSP1^High^ PDGFRβ^Low^,

CAF-S4: FAP^Neg-Low^ CD29^High^ αSMA^Med^ PDPN^Neg^ CD90^Med-High^ FSP1^Neg^ PDGFRβ^Med-High^ and

CAF-S5: FAP^Med^ CD29^Med^ αSMA^Neg-Low^ PDPN^Med^ CD90^Low^ FSP1^Low^ PDGFRβ^Med^ .

Dimensionality reduction by uniform manifold approximation and projection (UMAP), showed that while there is some overlap between CAFs and NCL fibroblasts, there was a distinct distribution of subpopulations (Fig. 2g). CAF-S3 was significantly more prevalent in NCL samples and CAF-S2 was found in both NCL and tumour (Fig. 2h). In contrast CAF-S1, CAF-S4 and CAF-S5 were significantly enriched in tumour samples (Fig. 2h).

**Fig. 3:**
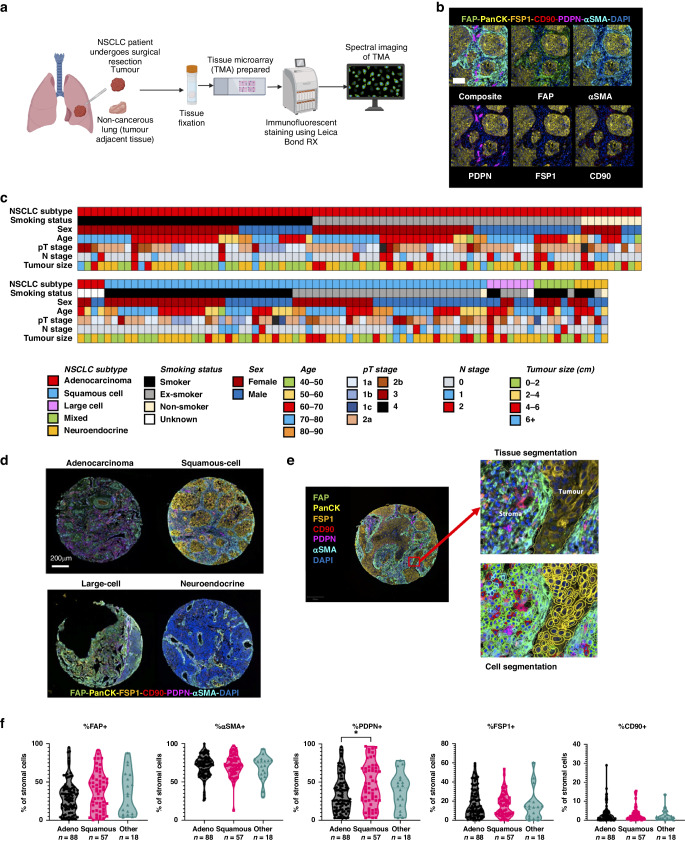
Multiplex immunofluorescence staining of CAFs in NSCLC. **a** Schematic showing the workflow of preparation of the TMA from NSCLC resections followed by immunofluorescent staining using a Leica Bond RX and then spectral imaging of slides; **b** Representative images showing the expression pattern of CAF markers FAP, αSMA, PDPN, FSP1 and CD90 relative to cancer cells identified by PanCK staining in a NSCLC tumour sample, scale bar 100 um; **c** Patient demographics of all patients in the TMA cohort; **d** Representative images of NSCLC subtypes adenocarcinoma, squamous cell carcinoma, large cell carcinoma and neuroendocrine carcinoma demonstrating heterogeneity between subtypes; **e** Images demonstrating the segmentation completed for each TMA core defining tumour and stromal regions by tissue segmentation and individual classification of cells by cell segmentation; **f** The percentage of stromal cells positive for CAF markers in different categories of NSCLC. Stats show Tukey’s multiple comparisons test results, *p ≤ 0.05.

To determine the relevance of these three subsets we investigated the spatial location and distribution of CAF subsets in NSCLC by multiplex immunofluorescent (MIF) staining of a microarray of 163 tumours using a tissue microarray (TMA) and spectral imaging (Fig. 3a). Tumour cores were stained with PanCK to identify tumour regions and the fibroblast markers FAP, PDPN, αSMA, FSP1 and CD90 were used to identify the key CAF subsets predominant in tumour tissue: CAF-S1, CAF-S4 and CAF-S5.

The MIF results showed clear staining of the fibroblasts markers in only the stromal regions, with the tumour regions stained by PanCK (Fig. 3b). As this was a diverse cohort, containing samples from multiple NSCLC subtypes (demographics of cohort shown in Fig. 3c), trends in marker expression were compared between subtypes (Fig. 3d, f) following segmentation and classification of cells (Fig. 3e). This revealed the level of heterogeneity between patients across subtypes, with the greatest range in expression levels shown in FAP and PDPN expression. PDPN expression also showed significant difference in expression levels between adenocarcinoma and squamous cell carcinoma, showing higher percentage positivity of PDPN in squamous cell carcinoma patients (Fig. 3f). CD90 staining was low, with very few cells classed as CD90^+^ across different classes of NSCLC. Consequently, CD90 was not be used to classify the CAF subsets in the MIF analysis. CAF-S1, CAF-S4 and CAF-S5 could still be identified independently of CD90 expression levels. The final definitions used for the MIF analysis were therefore: CAF-S1: FAP^ON^ αSMA^ON^ FSP1^OFF^ PDPN^ON^, CAF-S4: FAP^OFF^ αSMA^ON^ FSP1^OFF^ PDPN^OFF^, CAF-S5: FAP^ON^ αSMA^OFF^ FSP1^OFF^ PDPN^ON^.

**Fig. 4:**
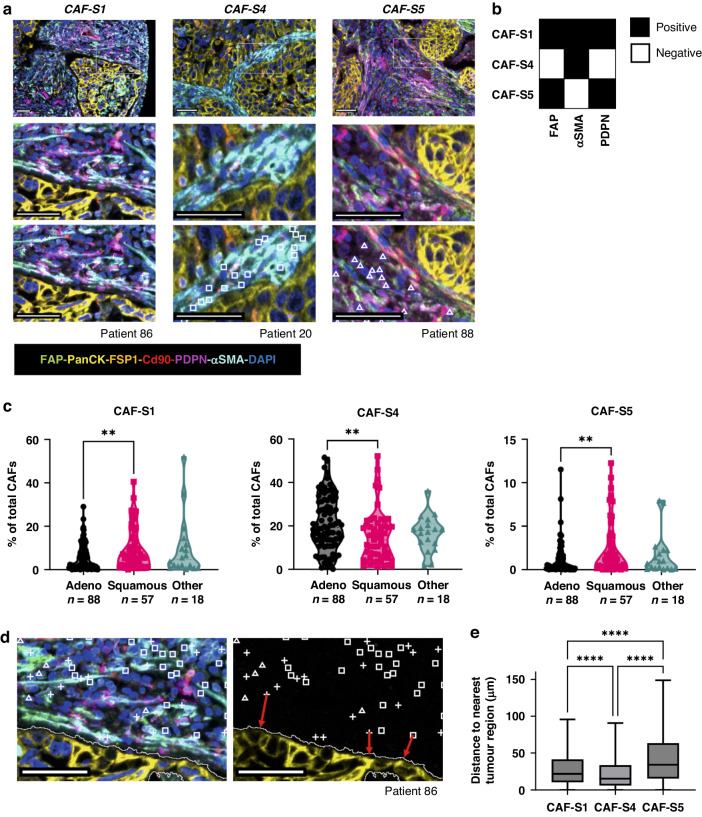
Spatial location of CAF subsets in NSCLC. **a** Representative images showing staining patterns and locations of CAFs from each subset analysed; **b** Schematic showing markers used to distinguish CAFs in each subset; **c** Percentage of total CAFs identified as belonging in each subset across NSCLC subtypes; **d** Representative image showing that CAFs were identified in QuPath and then the distance to the nearest tumour region (demonstrated by the red arrows) for each CAF was calculated; **e** Distances to the nearest tumour region of each cell classed in each subset across all 163 patients. Stats shown from Tukey’s multiple comparisons test, ****p < 0.0001, *n*_CAF-S1_ = 60,002, *n*_CAF-S4_ = 123,075, *n*_CAF-S5_ = 17,179. All scale bars shown are 50 μm.

CAFs were categorised into subsets depending on the markers they expressed (Fig. 4a) where the key markers used to distinguish each subset were FAP, αSMA and PDPN (Fig. 4b). We investigated whether distinct subsets dominated in different types of NSCLC by calculating the percentage of total CAFs (defined as those stained by any combination of the CAF markers investigated) of each CAF subset for adenocarcinoma, squamous cell carcinoma and other NSCLC subtypes (Fig. 4c). CAF-S1 and CAF-S5 were both upregulated in squamous cell carcinoma compared to adenocarcinoma, whereas CAF-S4 was upregulated in adenocarcinoma. We first considered whether there was a spatial difference between the subtypes, as we had previously observed visually that αSMA staining was dominant near tumour regions (Fig. 3b), and the key difference between the two subtypes is the lack of αSMA expression on CAF-S5 compared to CAF-S1. The spatial distribution was quantified by calculating the distance from each CAF to the nearest tumour region (Fig. 4d). This showed that CAF-S5 were more likely to be found further from tumour regions than CAF-S1, while CAF-S4 were found closest to tumour regions (Fig. 4e).

### CAF-S1 and CAF-S5 are functionally distinct

**Fig. 5:**
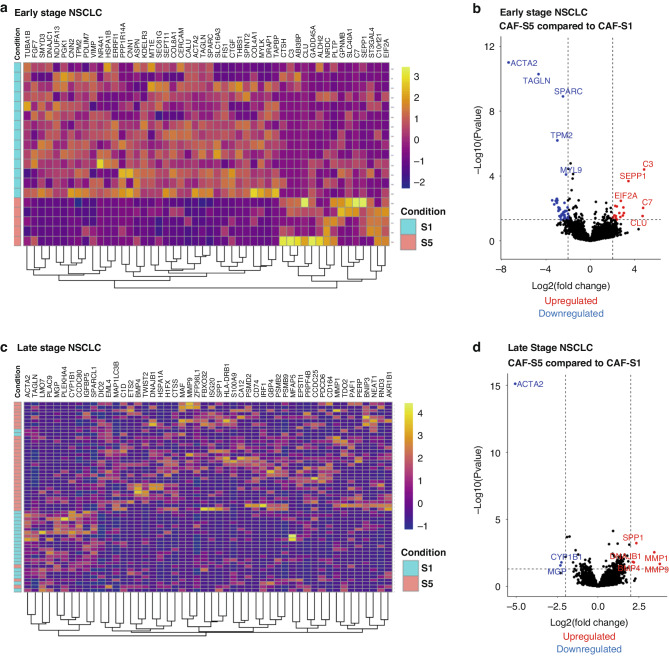
Functional analysis of CAF-S1 and CAF-S5 in early and late NSCLC. **a** Heat map showing top 50 differentially expressed genes in early NSCLC when comparing CAF-S1 and CAF-S5; **b** Volcano plot showing the most significantly up and downregulated genes when comparing CAF-S5 to CAF-S1 in early NSCLC; **c** Heat map showing top 50 differentially expressed genes in late NSCLC when comparing CAF-S1 and CAF-S5; **d** Volcano plot showing the most significantly up and downregulated genes when comparing CAF-S5 to CAF-S1 in late NSCLC. Early NSCLC data from Lambrechts et al. [22] and late NSCLC data from Wu et al. [34].

To characterise the differences in gene expression between FAP^+^ and PDPN^+^ subsets CAF-S1 and CAF-S5, single cell RNA sequencing data for early and late NSCLC, available from Lambrechts et al. [22] and Wu et al. [34] respectively was analysed. A heat map of the top 50 differentially expressed genes, showed CAF-S1 and CAF-S5 clustered separately for early NSCLC (Fig. 5a) and the majority cluster in late NSCLC (Fig. 5c). The most downregulated genes in CAF-S5 versus CAF-S1 in early NSCLC included TAGLN (transgelin), TPM2 (tropomyosin 2), SPARC (secreted protein acidic and cysteine rich) and MYL9 (myosin light chain 9) (Fig. 5b, normalised counts shown in Fig. S6). Genes upregulated in CAF-S5 included C3 (complement C3), SEPP1 (selenoprotein P), C7 (complement C7) and CLU (clusterin). In the case of late NSCLC, we see less significantly downregulated genes, but matrix metalloproteinases (MMP) genes for MMP1 and MMP9 were upregulated (Fig. 5d, normalised counts shown in Fig. S7). We also compared transcriptional differences between these subsets and the CAF-S4 subset (shown in Fig. S9) demonstrating distinct transcriptional differences between them.

### Presence of CAF-S1 or CAF-S5 correlates with worse 5 year survival in NSCLC

**Fig. 6:**
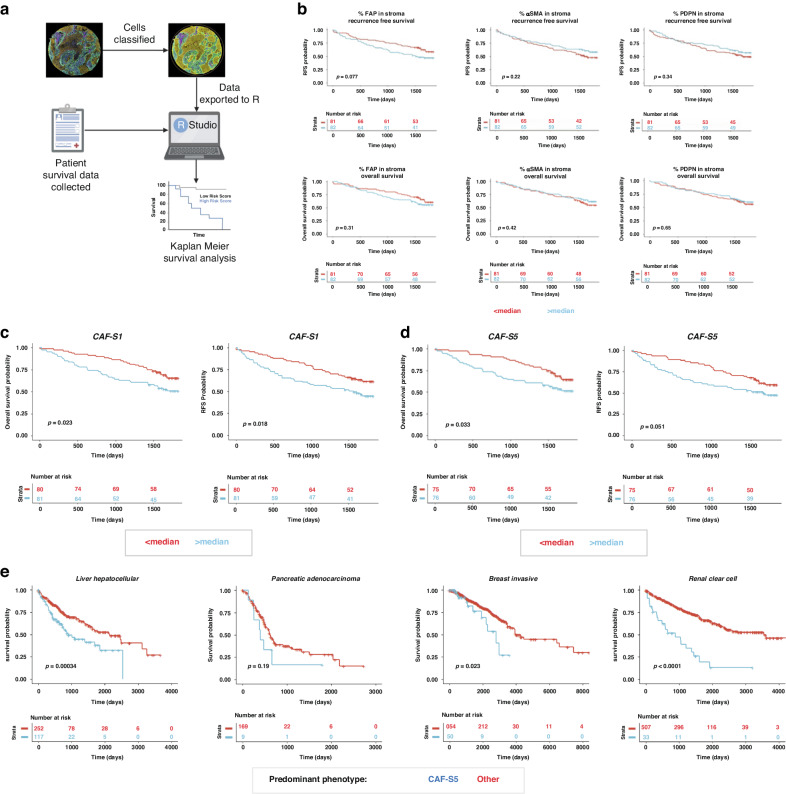
Survival analysis of CAF-S1 and CAF-S5 in NSCLC and other solid organ cancers. **a** Workflow of preparing data for survival analysis; **b** Survival looking at the percentage expression of FAP, PDPN and αSMA individually in the stroma, comparing above and below median expression. Both recurrence free survival and overall survival data shown; **c** Survival analysis (recurrence free and overall) of CAF-S1 when the proportion of the CAF subset present is greater or less than the median proportion expressed across all 163 patients; **d** Survival analysis (recurrence free and overall) of CAF-S5 when the proportion of the CAF subset present is greater or less than the median proportion expressed across all 163 patients; **e** Survival in other cancers from the TCGA dataset where each patient is defined as displaying a predominant phenotype by looking at FAP, PDPN and αSMA expression.

To understand if these subsets have prognostic significance, we performed survival analysis on our results from 163 NSCLC tumours, looking at whether the presence of CAF-S1 and CAF-S5 correlated with survival using the cell classifications from the TMA cohort (Fig. 6a). Analysis of the CAF markers alone did not reveal any significant effects on survival probability (Fig. 6b), although presence of FAP above median levels did demonstrate a trend towards poorer recurrence free survival. However, presence of either CAF-S1 or CAF-S5 was correlated with worse 5-year overall survival (Fig. 5c, d, cox regression analysis for survival shown in Supplementary Fig. S4). Presence of the CAF-S4 subset demonstrated a non-significant trend towards improved overall survival (Fig. S8).

### CAF-S5 signature indicates poorer survival in other cancers

Using the TCGA dataset we analysed the survival of patients who we expect to have greater prevalence of the novel CAF-S5 (based on bulk high expression of FAP and PDPN in the patient and low αSMA) across four solid organ cancers: liver hepatocellular, pancreatic adenocarcinoma, breast invasive carcinoma and renal clear cell carcinoma (Fig. 6e, cox regression analysis shown in Supplementary Fig. 4C). This revealed that the presence of these markers indicating CAF-S5 correlated with poor survival probability across these cancers, demonstrating conserved prognostic relevance of the CAF-S5 subset.

## Discussion

We have identified that in NSCLC, CAFs present as a heterogeneous population which can be divided into subsets depending on their expression levels of fibroblast activation markers. This heterogeneity of CAFs is found both between and within patient samples. Two of the subsets, CAF-S2 and CAF-S3, express low levels of markers used to identify activated fibroblasts. This, and the finding that there are more CAF-S3 in NCL and an equal presence of CAF-S2 in NCL and tumour, suggests that these subsets are representative of more quiescent lung fibroblasts found in health. Conversely, CAF-S1, CAF-S4 and CAF-S5 are more prevalent in tumour tissue. CAF-S5 is a novel subset, identified here as expressing FAP^Med^ CD29^Med^ αSMA^Neg-Low^ PDPN^Med^ CD90^Low^ FSP1^Low^ and PDGFRβ^Med^. These subsets express different levels of fibroblast activation markers, demonstrating that no single fibroblast marker can be used to isolate CAFs in NSCLC. This suggests that studies targeting single markers to deplete CAFs are targeting limited subsets, and our work demonstrates that they need to be considered together.

These fibroblast markers can also be used to identify CAF subsets through multiplex immunofluorescence imaging when the definitions outlined from the flow cytometry analysis are converted to binary definitions. Although binary definitions limited the ability to classify as thoroughly as the flow cytometry analysis, they allowed for confident assigning of CAFs to their respective subsets as described. The three subsets identified as more prevalent in the tumour (CAF-S1, CAF-S4 and CAF-S5) were investigated by staining for CAF markers FAP, αSMA, PDPN, CD90 and FSP1 in a cohort of 163 patients that were part of a TMA. A limitation of the study is the use of a TMA. We assessed 1 mm cores of tissue from each patient and others have demonstrated this method can successfully predict patient outcome post surgical resection in lung adenocarcinoma [37]. Assessing the distribution of each marker across different tissue classes revealed differences between adenocarcinoma and squamous cell carcinoma, notably the expression of PDPN being higher in squamous cell carcinoma. The expression of PDPN has been linked to poor prognosis in cancer, and is hypothesised to play roles in invasion, epithelial to mesenchymal transition (EMT) and metastasis [38, 39]. PDPN positivity correlates with greater invasiveness in lung adenocarcinomas [40] and functionally CAFs expressing PDPN and FAP have previously been identified to suppress T cell proliferation in a nitric oxide dependent manner in breast cancer [41]. It would therefore be expected PDPN + CAF subsets (CAF-S1 and CAF-S5) would be associated with poorer long-term survival, and this was confirmed in our study.

Comparing the proportions of CAF subsets between NSCLC subtypes, we observed a higher proportion of CAF-S1 and CAF-S5 in squamous cell carcinoma, and a higher proportion of CAF-S4 in adenocarcinoma. This distribution is likely due to the expression of PDPN in CAF-S1 and CAF-S5 as previously discussed. This finding is supported by studies in other cancers, as CAF subsets in breast cancer have been shown to be dependent on histology classification, with different tumour classes presenting enrichment with different CAF subsets [42]. Recent studies suggest that this may be due to a reservoir of fibroblasts in healthy tissues, which are capable of activation to various phenotypes dependent on disease state [43].

Other groups have investigated FAP and αSMA in NSCLC, often using single makers in IHC, and have found various prognostic features. This includes FAP^+^ CAFs to be an indicator of positive outcome in squamous cell carcinoma [44] and the same group also suggested in a subsequent study that this was due to high infiltration of CD8 and CD3 positive T cells in the tumours [45]. Conversely another study identified FAP^+^ CAFs and associated them with poor prognosis in adenocarcinoma, particularly in the presence of low CD8 T cell infiltration in the stroma [46]. In this study we found that using a single marker may not determine prognostic significance, but rather there are CAF phenotypes which can be identified using a combination of markers. This approach was also used by another group which utilised multiplex staining of FAP, αSMA, PDGFRα and PDGFRβ by immunofluorescence, identifying CAFs of each combination of the markers used [47]. Although a direct comparison cannot be made as the same makers were not used, here a poor prognosis subset (CAF7) broadly aligns with our CAF-S1 subset and favourable prognosis (CAF13) broadly with CAF-S4 subset (Supplementary Fig. S8). A recent study identified CAF subsets in NSCLC by utilising multiplexed imaging mass cytometry and included the key markers we used [48]. Although their analysis does not allow direct comparison to our study to identify CAF-S1 or CAF-S5, they did identify a favourable outcome with SMA CAFs which align with our CAF-S4. These studies further highlight the novelty of the CAF-S5 subset identified here.

To further characterise differences between CAF-S1 and CAF-S5 we analysed two single cell RNA sequencing datasets for NSCLC, published by Lambrechts et al. [22] and Wu et al. [34]. We primarily focussed on these subsets due to their association with poor prognosis. Subsets of fibroblasts defined as CAF-S1 or CAF-S5 by our established criteria were compared. As the defining difference between the two subsets is the expression of αSMA, the main predicted difference was that CAF-S5 would not be a contractile phenotype. This was further confirmed by the finding that genes such as TAGLN and TPM2 were downregulated in CAF-S5 in early NSCLC, as they would contribute to contractility also, and that contractile pathways were suppressed (Fig. S5). The upregulation of complement genes C3 and C7 suggests that CAF-S5 are an inflammatory subset. The upregulation of C3 in immunomodulatory stromal cells has previously been identified in mouse and human studies, showing that these cells are capable of influencing the tumour immune response [49]. Previously described immunomodulatory subsets have demonstrated high levels of chemokines such as CXCL12, further indicating their role in the influence of the immune system and inflammatory response [50]. In late NSCLC downregulation of contractile genes such as TAGLN were also observed, but others such as MMP1, MMP9 and SPP1 were found to be upregulated, which have functions in matrix remodelling and invasion and are linked to poor prognosis in lung cancer [51, 52]. This suggested that the CAF-S5 subset could promote disease proliferation, metastasis and resistance to therapy, which would contribute to worse overall survival.

These findings in NSCLC suggest an alignment with the CAF breakdown previously reported in pancreatic cancer of iCAF and myCAF, where iCAF represent an inflammatory subset and myCAF a myofibroblastic one [17]. In pancreatic cancer myCAF were found in close proximity to cancer cells compared to the more distally located iCAF. We find in NSCLC, CAF-S5 were also located more distally from tumour cells, suggesting they are more likely induced by secreted factors rather than requiring cell contact for interactions. This is supported by a separate study in NSCLC where CAFs expressing FAP and αSMA were also shown to be located closer to tumour nests than those only FAP^+^ [23]. Therefore, our work supports these findings in NSCLC, but also we demonstrate both subsets contribute to poor survival outcome.

To further understand the influence of the CAF-S5 subset on survival in other cancers we analysed the TCGA dataset for multiple solid organ cancers (liver, pancreatic, breast and renal clear cell). For these cancers investigated, this showed decreased survival probability when CAF-S5 was dominant, compared to all other patients in the cohort. It has previously been shown that patients expressing the CAF-S1 phenotype in breast cancer have increased survival probability compared to other groups [20]. Our analysis suggests the presence and prevalence of the CAF-S5 subset in breast cancer warrants further investigation. By not considering the CAF-S5 subset in patients, and only considering the CAF-S1, patient outcome may be incorrectly predicted. This highlights the importance of the novel CAF-S5 subset as a predictor of poor outcome.

### Supplementary information

Supplementary Information

## Data Availability

Data used in this study are available from the corresponding author upon reasonable request.

## Code availability

Codes used in this study are available from the corresponding author upon reasonable request.
