# Supplementary material for: Cancer-associated fibroblasts expressing fibroblast activation protein and podoplanin in non-small cell lung cancer predict poor clinical outcome
Source: Br J Cancer. 2024 Apr 6;130(11):1758–69. doi: 10.1038/s41416-024-02671-1 (PMC11130154; doi:10.1038/s41416-024-02671-1)
Supplement: Supplementary file 1 — Supplementary Information [file 41416_2024_2671_MOESM1_ESM.docx]

**Supplementary Information**

Cancer-associated fibroblasts expressing fibroblast activation protein and podoplanin in non-small cell lung cancer predict poor clinical outcome

Layla Mathieson, Lilian Koppensteiner, David A Dorward, Richard A O’Connor, Ahsan R Akram^.^

**Table S1: Antibodies used for flow cytometry staining.**

| Marker | Colour | Supplier | ul/test | Catalogue No. | Isotype | ul/test | Iso Catalogue No. |
| --- | --- | --- | --- | --- | --- | --- | --- |
| CD45 | BV605 | BioLegend | 5 | 368524 | IgG1 M | 5 | 400161 |
| CD31 | BV605 | BioLegend | 5 | 303122 | IgG1 M | 5 | 400161 |
| EpCAM | BV605 | BioLegend | 5 | 324224 | IgG2a M | 5 | 400349 |
| CD90 | VioBlue | Miltenyi | 2 | 130-119-890 | IgG1 M | 2 | 130-113-767 |
| Zombie | UV | BioLegend | 1 | 423108 | NA | NA | NA |
| FAP | APC | R&D | 5 | FAB3715A | IgG1 M | 5 | IC002A |
| PDGFRβ | AF594 | R&D | 5 | FAB1263T | IgG1 M | 5 | IC002T |
| CD29 | AF488 | BioLegend | 5 | 303016 | IgG1 M | 5 | 400129 |
| PDPN | APC-Cy7 | BioLegend | 5 | 337030 | IgG2a R | 2.5 | 400524 |
| αSMA | AF750 | R&D | 5 | IC1420S | IgG2a M | 5 | IC 003S |
| FSP-1 | PE | BioLegend | 5 | 370004 | IgG1 M | 5 | 400139 |

**Table S2: Multiplex immunofluorescence antibodies used and their OPAL pairings.**

| Primary Antibody | Catalogue No. | Antibody Dilution | OPAL Pairing | OPAL Dilution | Staining Position |
| --- | --- | --- | --- | --- | --- |
| FAP | Ab207178 | 1:100 | OPAL 520 | 1:150 | 1 |
| CD90 | Ab92574 | 1:50 | OPAL 620 | 1:100 | 2 |
| FSP1 | Ab197896 | 1:4000 | OPAL 570 | 1:150 | 3 |
| PDPN | Ab236529 | 1:4000 | OPAL 650 | 1:150 | 4 |
| αSMA | Ab124964 | 1:1000 | OPAL 690 | 1:150 | 5 |
| PanCK | Ab27988 | 1:200 | OPAL 540 | 1:150 | 6 |


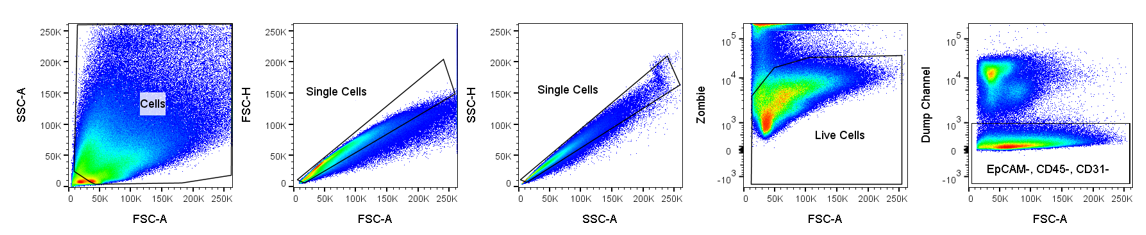


**Figure S1:** **Gating strategy used to identify fibroblasts.** Fibroblasts were defined as single, live cells which were EpCAM, CD45 and CD31 negative.


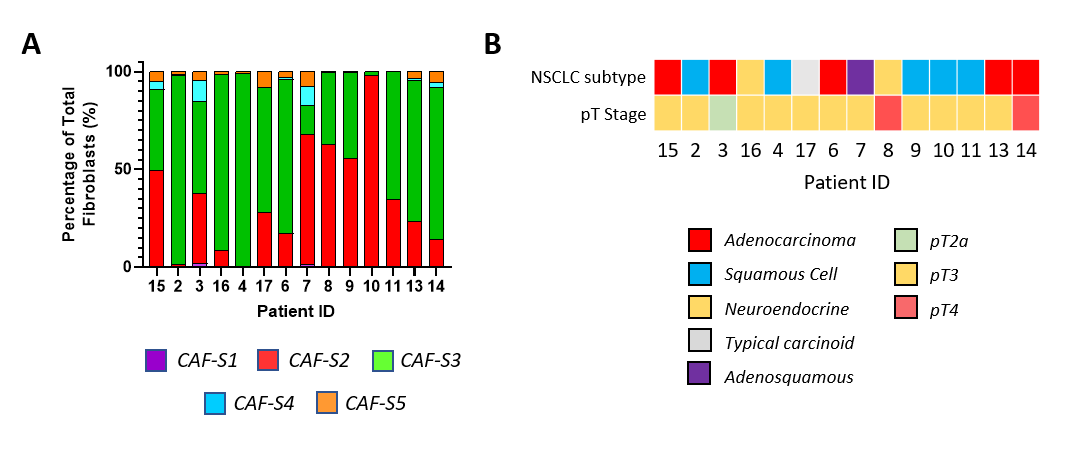


**Figure S2: CAF subset distribution in NCL samples.** (A) CAF subset proportions identified in NCL samples from patients undergoing lung cancer resections; (B) Patient demographics for each sample showing their lung cancer subtype and pT stage.


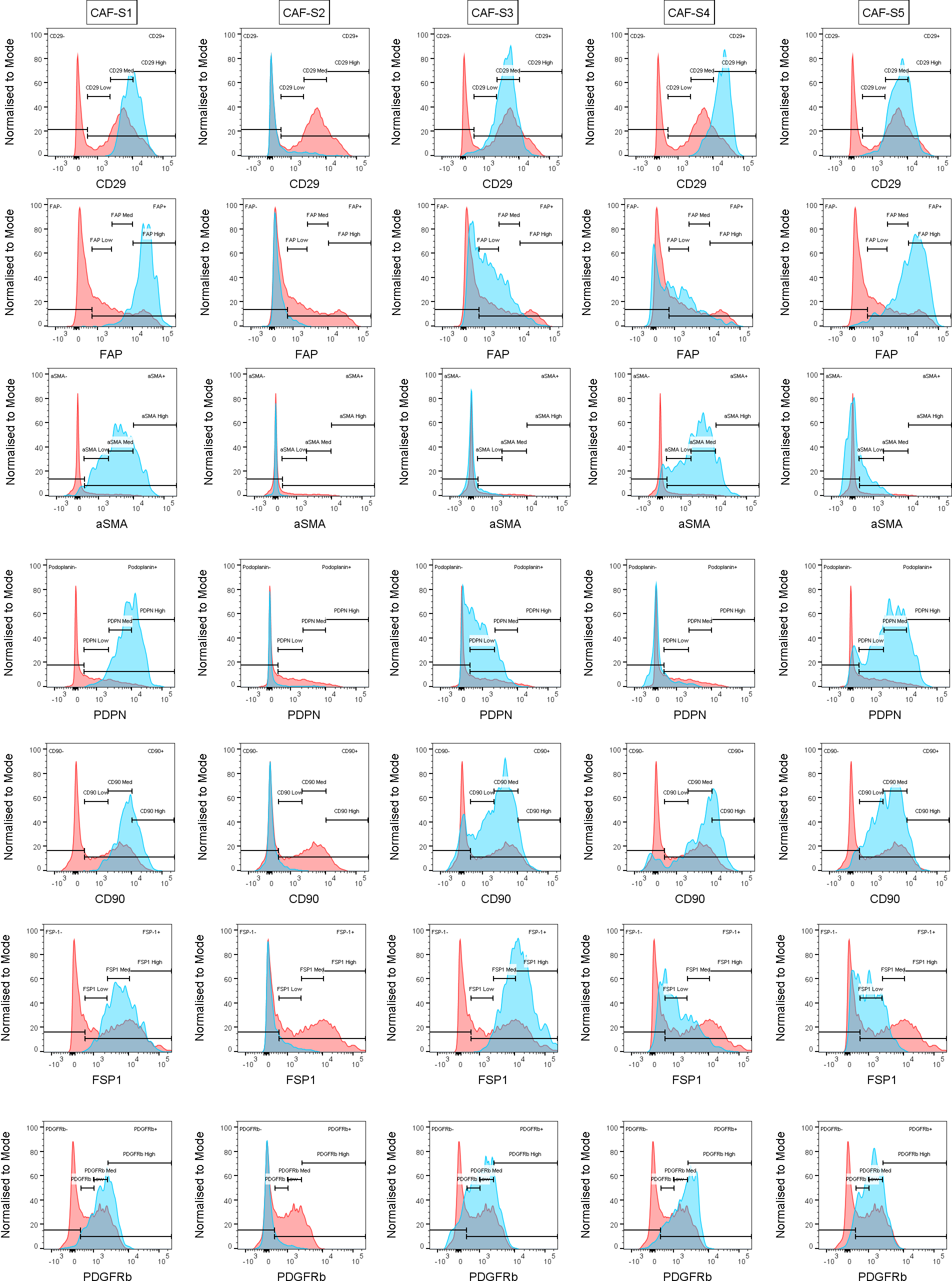


**Figure S3: Classification of low, intermediate or high populations.** Histograms showing the expression of each marker in each subset and the definitions used to determine if levels were low, medium or high expression. Pink histograms show all data for all cells in analysis and blue shows just those cells classed as each subset.

**
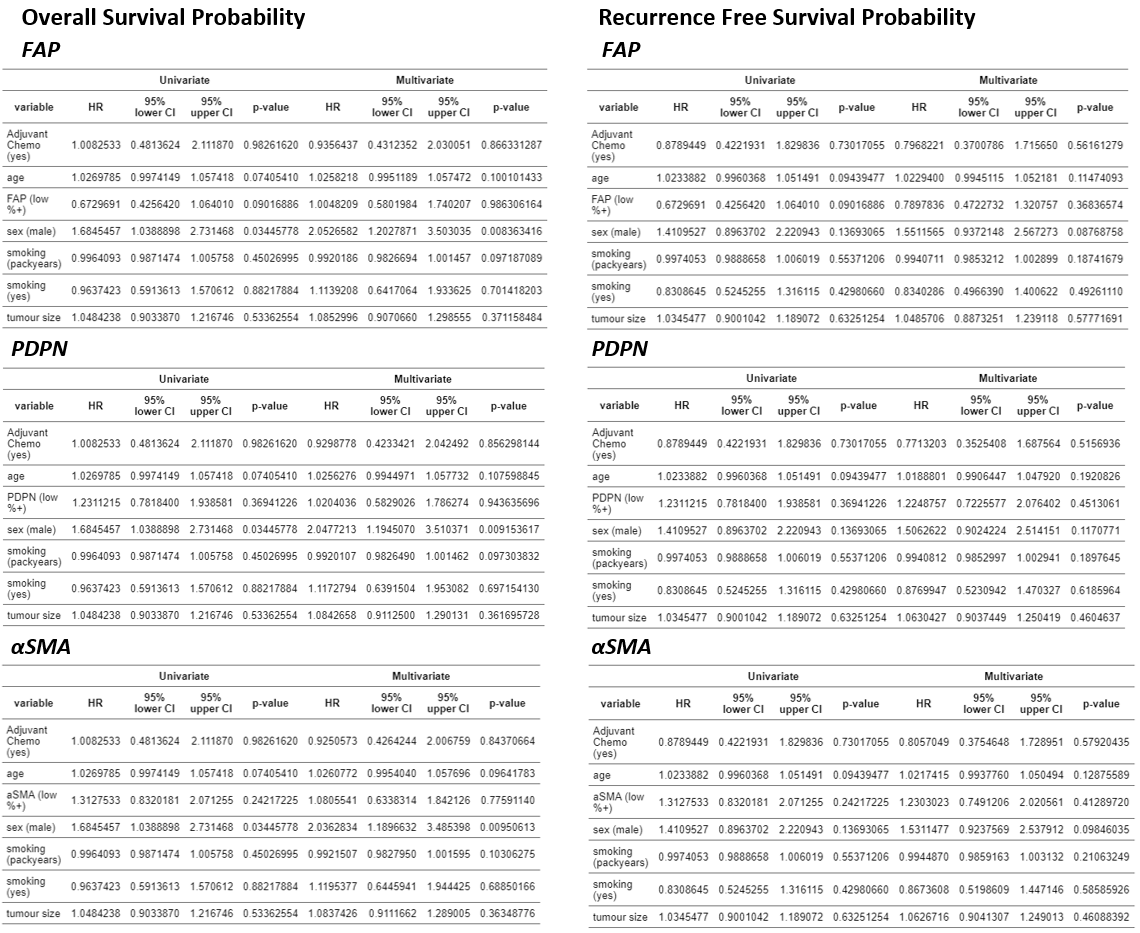
**

**Figure S4A: Cox regression analysis for assessment of impact of individual markers on survival outcome.** Analysis was corrected for patient age, sex, smoking (pack years), marker expression, adjuvant chemotherapy and tumour size.

**
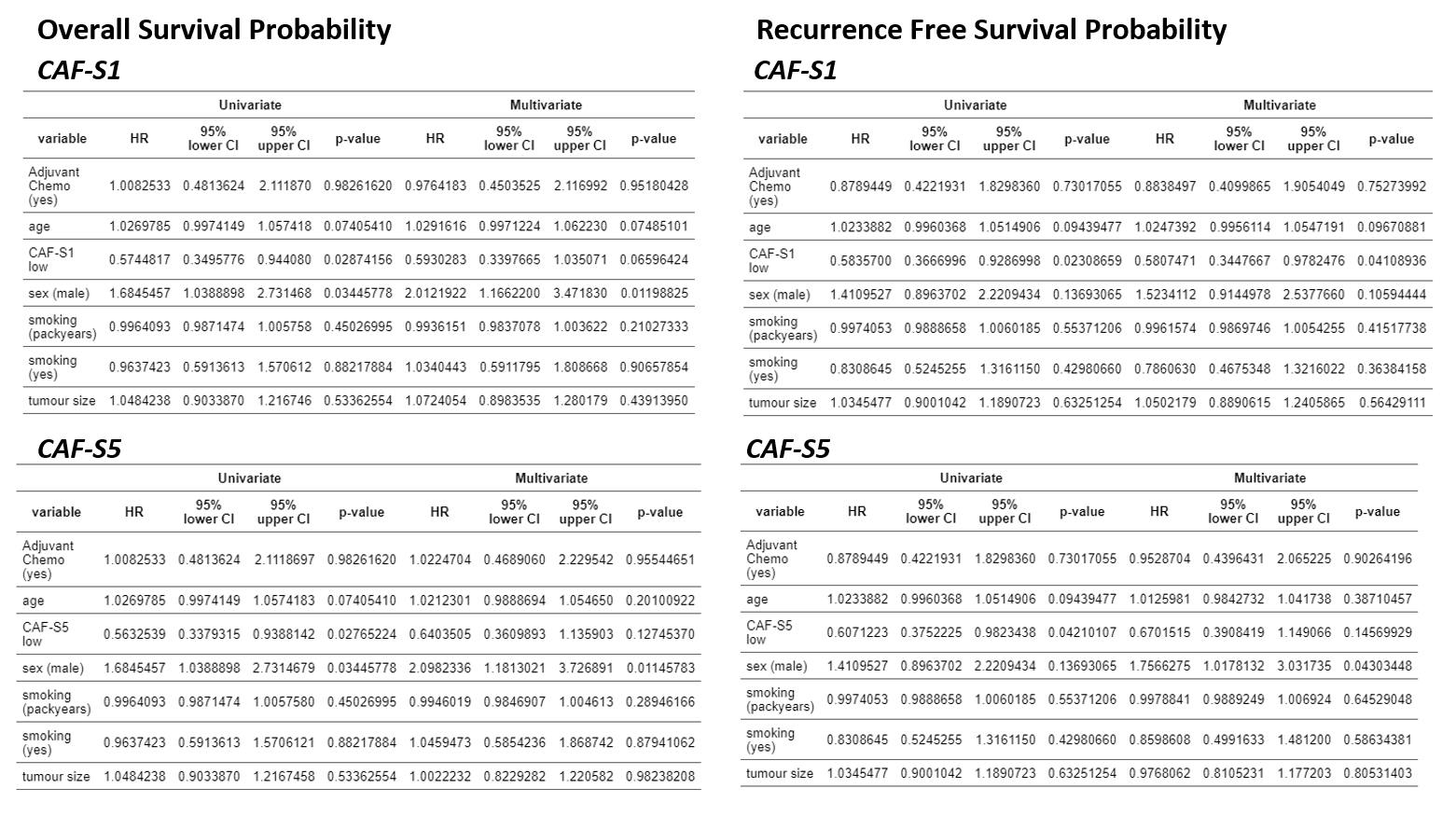
**

**Figure S4B: Cox regression analysis for assessment of impact of CAF subsets on survival outcome.** Analysis was corrected for patient age, sex, smoking (pack years), subset expression, adjuvant chemotherapy and tumour size.

**
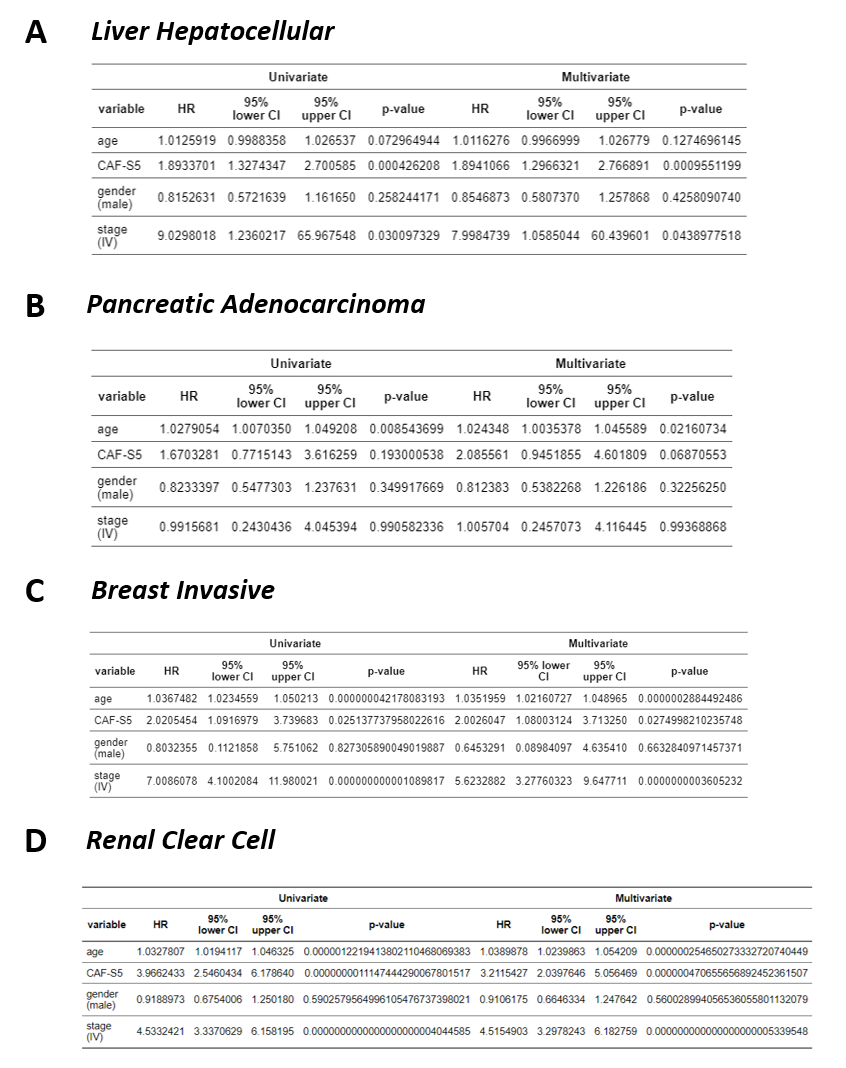
**

**Figure S4C: Cox regression analysis for assessment of impact of CAF-S5 subset on survival outcome for TCGA datasets analysed.**

**
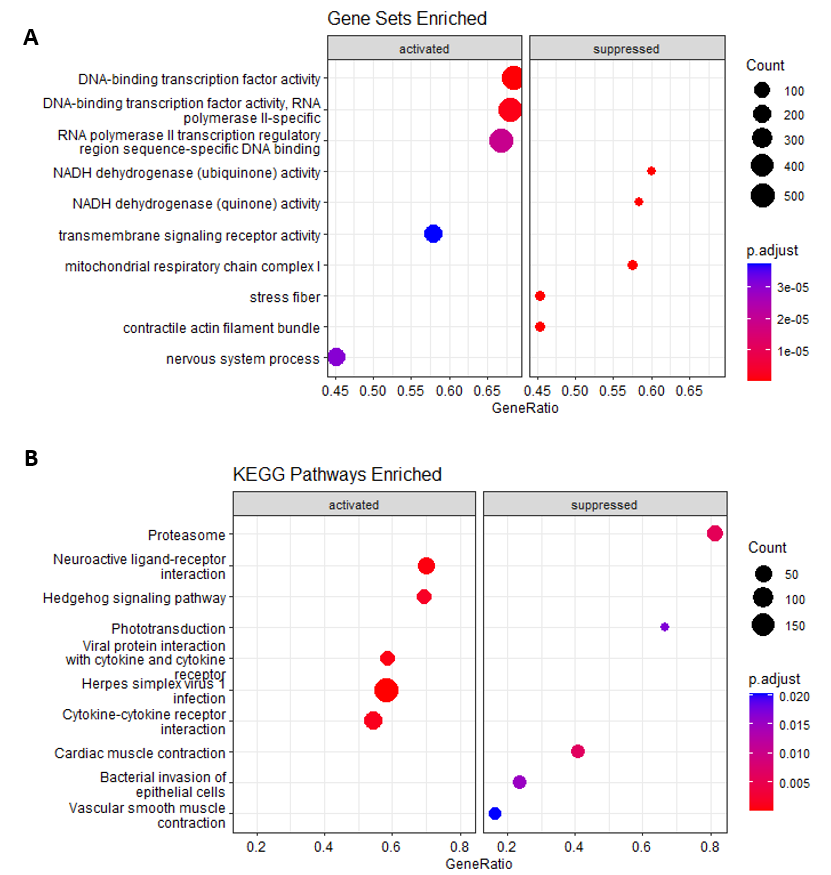
**

**Figure S5: Gene set and pathways identified as enriched in Lambrechts et al. early NSCLC data.** (A) Gene sets activated and suppressed in CAF-S5 compare to CAF-S1 following gene set enrichment analysis; (B) KEGG pathways activated and suppressed in CAF-S5 compared to CAF-S1 following KEGG pathway analysis.

**
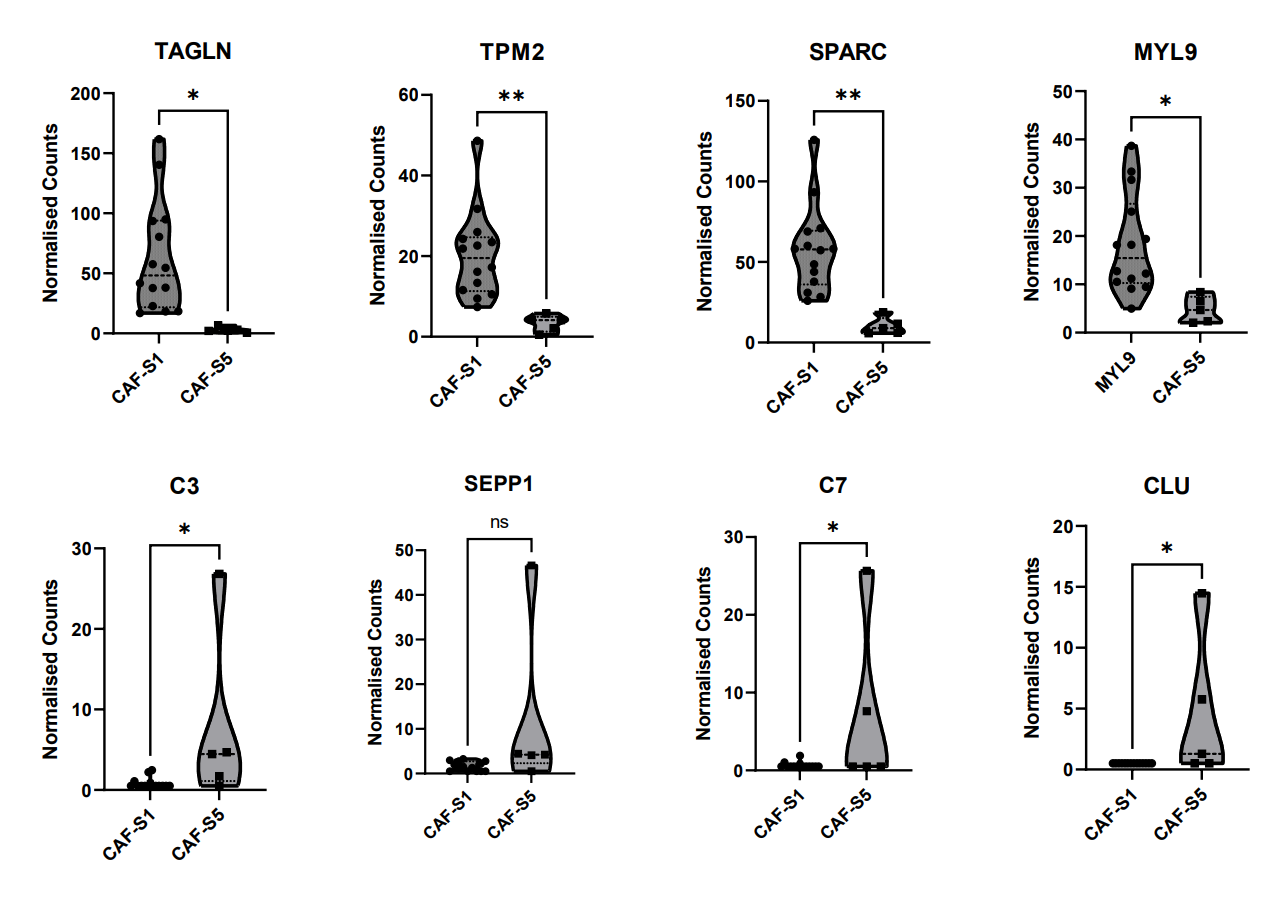
**

**Figure S6: Most significantly differentially expressed genes when comparing CAF-S1 to CAF-S5 in single cell dataset from early NSCLC in Lambrechts et al.**

**
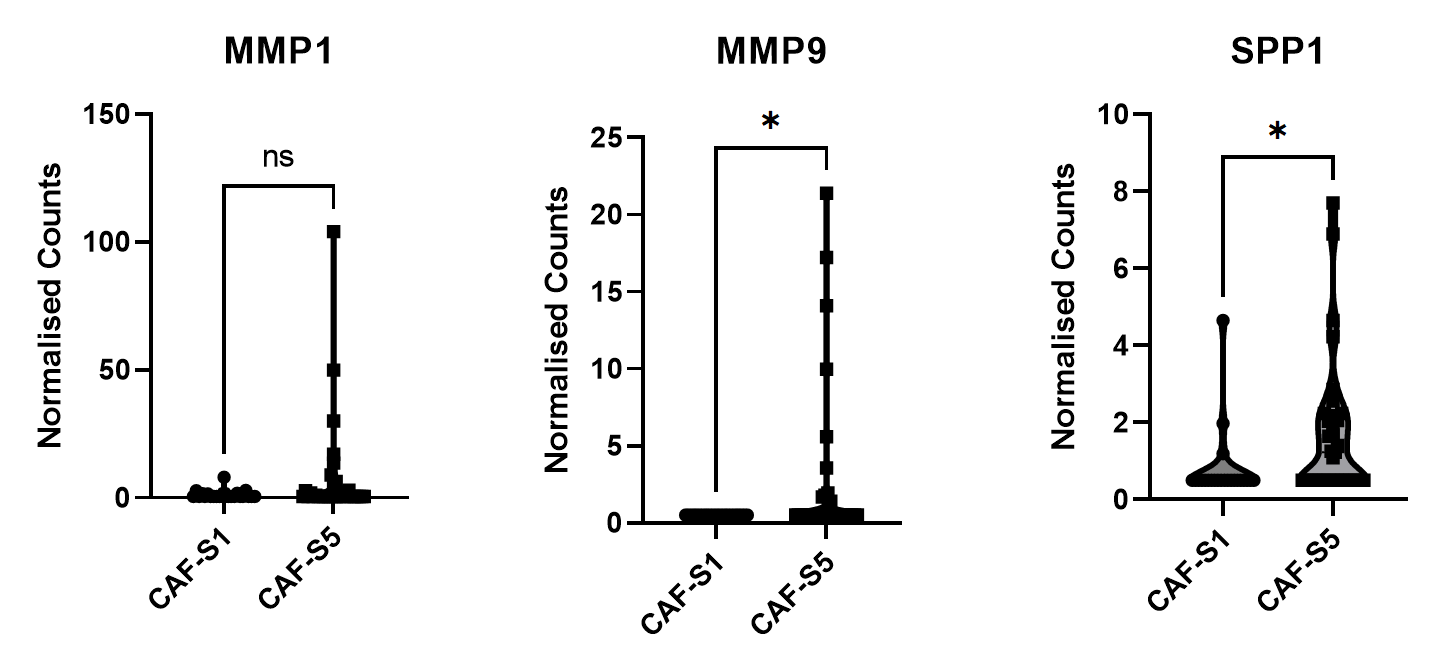
**

**Figure S7: Most significantly differentially expressed genes when comparing CAF-S1 to CAF-S5 in single cell dataset from late NSCLC in Wu et al.**

**
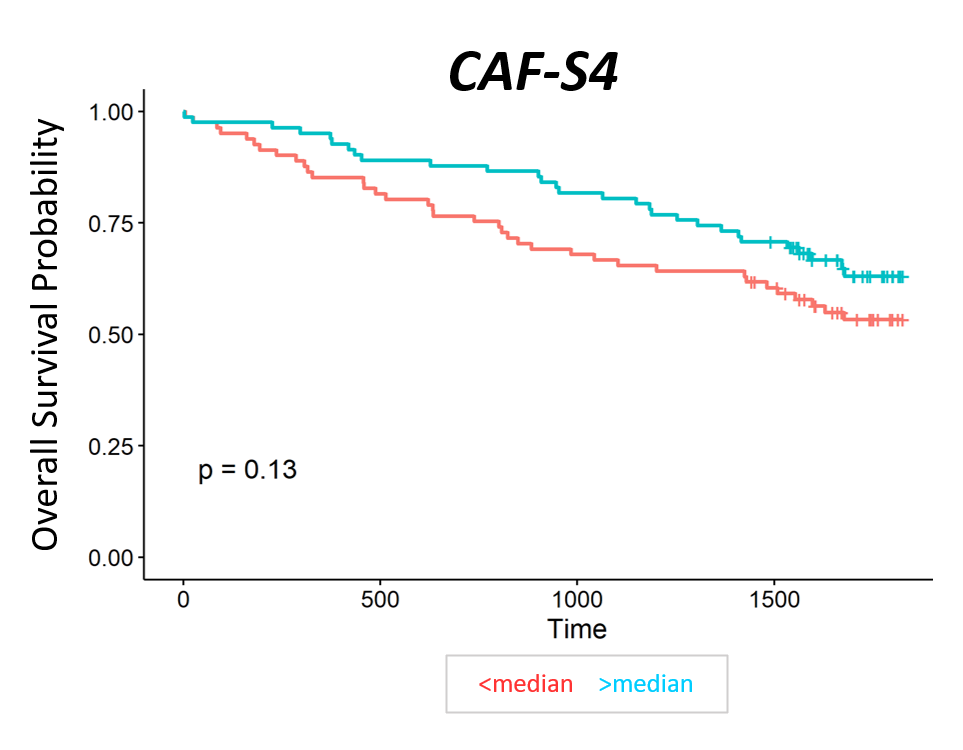
**

**Figure S8: The overall survival probability of patients expressing the CAF-S4 phenotype above and below median expression in the cohort.**


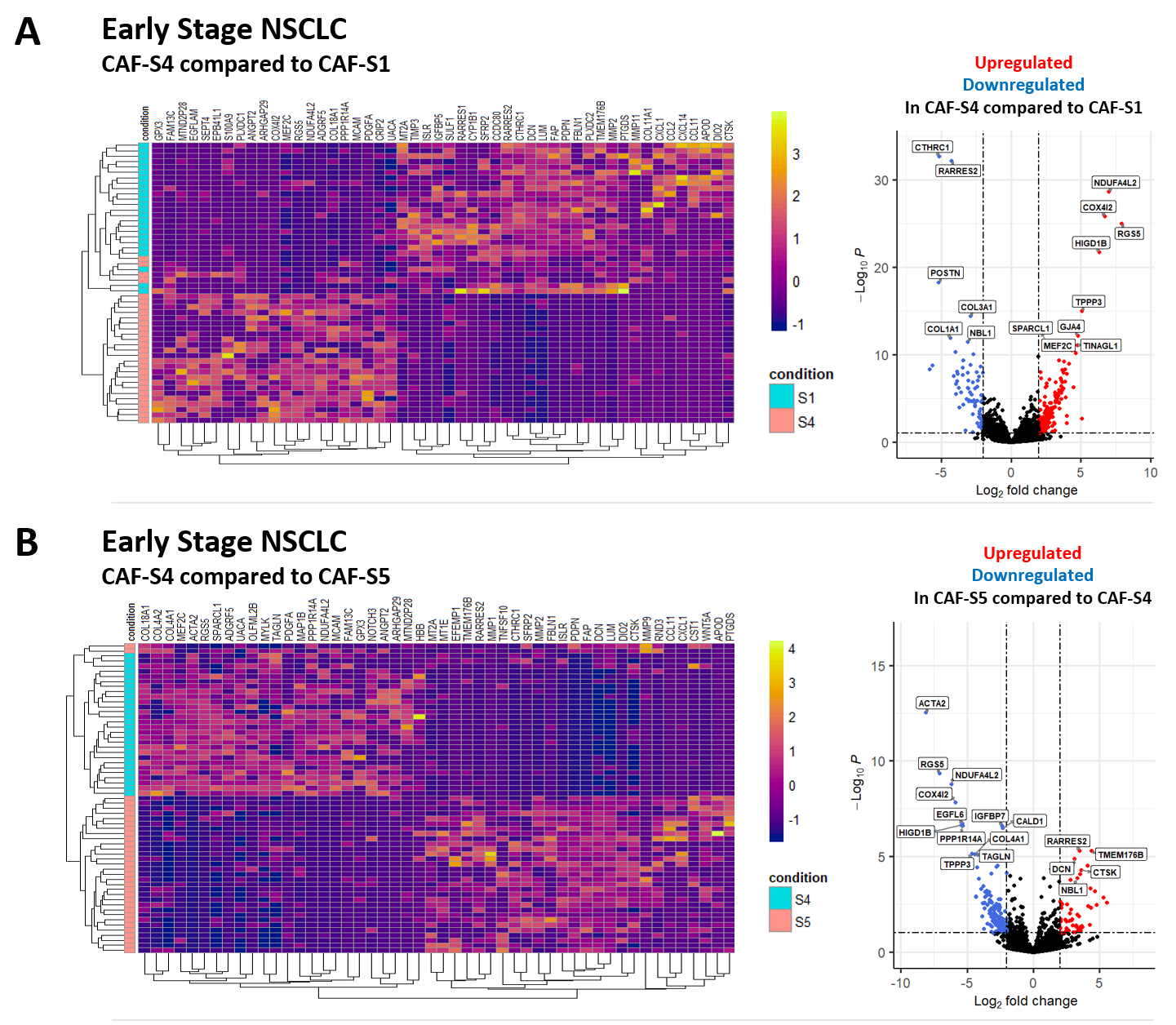

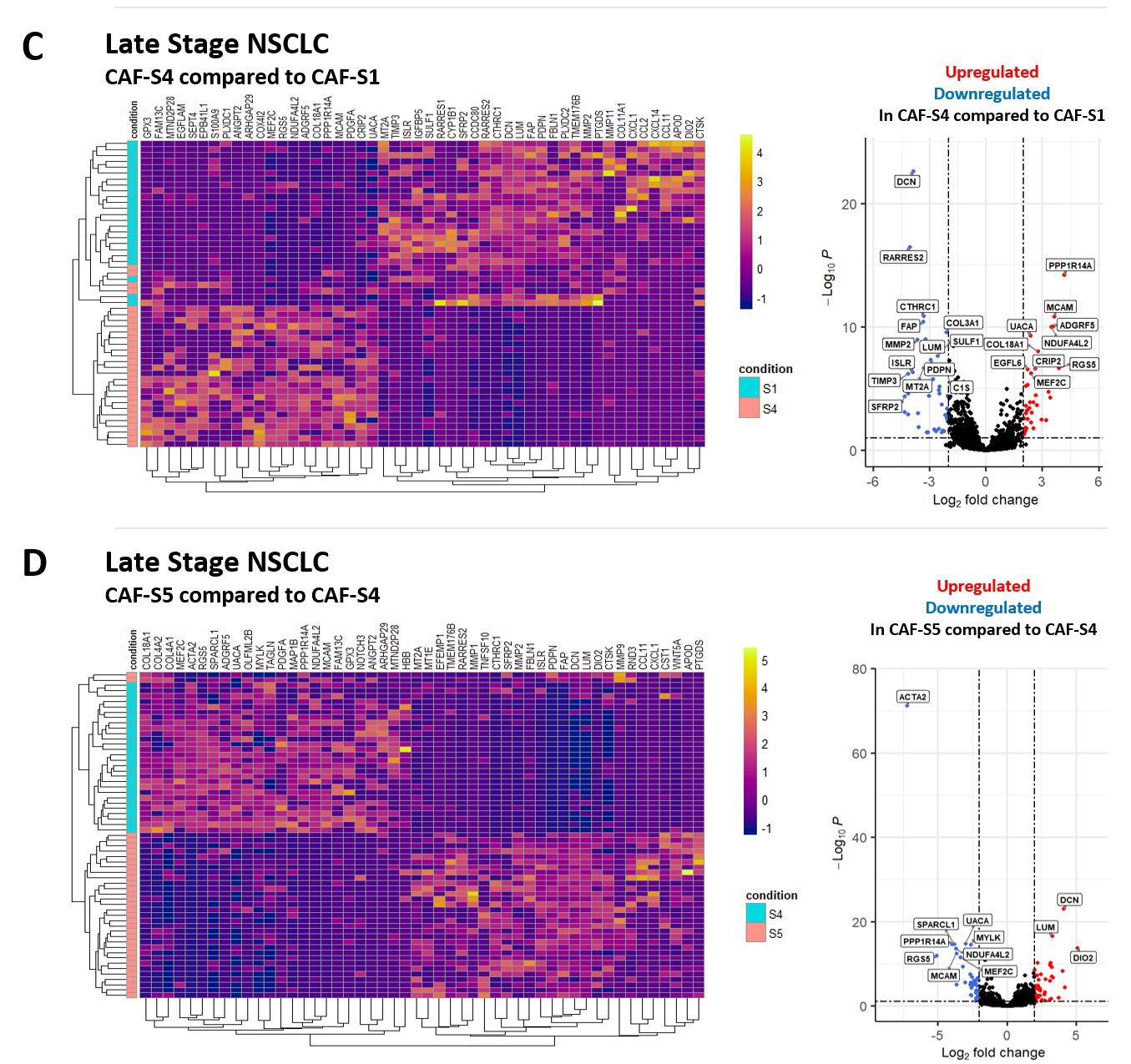


**Figure S9: Single cell RNA sequencing data comparing the CAF-S4 subset to CAF-S1 and CAF-S5 utilising the early NSCLC dataset from Lambrechts et al. and the late NSCLC dataset from Wu et al.**

**Table S3: Patient demographic information for all samples analysed for flow cytometry analysis of CAF subsets.**

| **Patient** | **Age** | **Sex** | **Smoking status at Diagnosis** | **Histology** | **Grade** | **Max Diameter (mm)** | **Background lung** | **pTN staging** |
| --- | --- | --- | --- | --- | --- | --- | --- | --- |
| 1 | 75 | F | Ex-smoker | Adenocarcinoma- lepidic predominance | poorly differentiated | 46 | infarct and pigment laden macrophages | pT2b N0 |
| 2 | 66 | F | Ex-smoker | Squamous Cell Carcinoma | moderately differentiated | 65 | unremarkable | pT3 N1 |
| 3 | 68 | F | Ex-smoker | Adenocarcinoma- solid predominance | poorly differentiated | 38 | emphysema | pT2a N0 |
| 4 | 71 | M | Non-smoker | Squamous Cell Carcinoma | poorly differentiated | 56 | granulomatous inflammation | pT3 N1 |
| 5 | 69 | F | Smoker | Squamous Cell Carcinoma | moderately differentiated | 67 | emphysema and chronic inflammation | pT3 N1 |
| 6 | 57 | M | Ex-smoker | Adenocarcinoma- lepidic predominance | well differentiated | 96 | no comment made | pT3 N0 |
| 7 | 83 | F | Non-smoker | Adeno-Squamous carcinoma (squamous cell predominant) | poorly differentiated | 60 | reactive and inflammatory changes | pT3 N1 |
| 8 | 80 | M | Ex-smoker | Large cell neuroendocrine (90%) adenocarcinoma (10%) | poorly differentiated | 95 | minor emphysema | pT4 N0 |
| 9 | 76 | F | Ex-smoker | Squamous Cell Carcinoma | - | 45 | scattered non specific fibrosis | pT3 N0 |
| 10 | 79 | M | Ex-smoker | Squamous Cell Carcinoma | poorly differentiated | 62 | unremarkable | pT3 N2 |
| 11 | 83 | M | Ex-smoker | Squamous Cell Carcinoma | moderately differentiated | 57 | unremarkable | pT3 N0 |
| 12 | 68 | M | Smoker | Adenocarcinoma- solid predominance | poorly differentiated | 55 | fibrosis background | pT3 N0 |
| 13 | 67 | F | Ex-smoker | Adenocarcinoma- papillary predominance | moderately differentiated | 32 | emphysema | pT3 N0 |
| 14 | 67 | M | Ex-smoker | Adenocarcinoma, non-mucinous lepidic and papillary patterns | moderately differentiated | 150 | emphysema | pT4 N1 |
| 15 | 64 | M | Ex-smoker | Adenocarcinoma- acinar predominance | moderately differentiated | 67 | unremarkable | pT3 N0 |
| 16 | 73 | F | Ex-smoker | Large cell neuroendocrine | poorly differentiated | 25 | patchy obstructive change, otherwise normal. | pT2a N1 |
| 17 | 72 | F | Ex-smoker | Typical carcinoid | well differentiated | 28 | Diffuse idiopathic pulmonary neuroendocrine cell hyperplasia background lung | pT1c N0 |
